# The Impact of 6 and 12 Months in Space on Human Brain Structure and Intracranial Fluid Shifts

**DOI:** 10.1093/texcom/tgaa023

**Published:** 2020-06-15

**Authors:** Kathleen E Hupfeld, Heather R McGregor, Jessica K Lee, Nichole E Beltran, Igor S Kofman, Yiri E De Dios, Patti A Reuter-Lorenz, Roy F Riascos, Ofer Pasternak, Scott J Wood, Jacob J Bloomberg, Ajitkumar P Mulavara, Rachael D Seidler, Alzheimer’s Disease Neuroimaging Initiative

**Affiliations:** Department of Applied Physiology and Kinesiology, University of Florida, Gainesville, FL 32608, USA; Department of Applied Physiology and Kinesiology, University of Florida, Gainesville, FL 32608, USA; German Aerospace Center (Deutsches Zentrum für Luft- und Raumfahrt), 51147 Cologne, Germany; KBR, Houston, TX 77002, USA; KBR, Houston, TX 77002, USA; KBR, Houston, TX 77002, USA; Department of Psychology, University of Michigan, Ann Arbor, MI 48109, USA; Department of Diagnostic and Interventional Imaging, University of Texas Health Science Center at Houston, Houston, TX 77030, USA; Departments of Psychology and Radiology, Brigham and Women’s Hospital, Harvard Medical School, Boston, MA 02115, USA; Neuroscience Laboratory, Biomedical Research and Environmental Sciences Division, NASA Johnson Space Center, Houston, TX 77058, USA; Neuroscience Laboratory, Biomedical Research and Environmental Sciences Division, NASA Johnson Space Center, Houston, TX 77058, USA; KBR, Houston, TX 77002, USA; Department of Applied Physiology and Kinesiology, University of Florida, Gainesville, FL 32608, USA; Department of Neurology, University of Florida, Gainesville, FL 32611, USA

**Keywords:** cortical thickness, free water, gray matter volume, spaceflight, ventricular volume

## Abstract

As plans develop for Mars missions, it is important to understand how long-duration spaceflight impacts brain health. Here we report how 12-month (*n* = 2 astronauts) versus 6-month (*n* = 10 astronauts) missions impact brain structure and fluid shifts. We collected MRI scans once before flight and four times after flight. Astronauts served as their own controls; we evaluated pre- to postflight changes and return toward preflight levels across the 4 postflight points. We also provide data to illustrate typical brain changes over 7 years in a reference dataset. Twelve months in space generally resulted in larger changes across multiple brain areas compared with 6-month missions and aging, particularly for fluid shifts. The majority of changes returned to preflight levels by 6 months after flight. Ventricular volume substantially increased for 1 of the 12-month astronauts (left: +25%, right: +23%) and the 6-month astronauts (left: 17 ± 12%, right: 24 ± 6%) and exhibited little recovery at 6 months. Several changes correlated with past flight experience; those with less time between subsequent missions had larger preflight ventricles and smaller ventricular volume increases with flight. This suggests that spaceflight-induced ventricular changes may endure for long periods after flight. These results provide insight into brain changes that occur with long-duration spaceflight and demonstrate the need for closer study of fluid shifts.

## Introduction

After nearly 60 years of manned space travel, there are many unknowns about the effects of spaceflight on the human brain. Understanding the impact of spaceflight on brain health is critical, given imminent plans to extend the duration and distance of human space travel. In the present work, we use the term “spaceflight” to refer to the entire flight duration, including travel to and from the International Space Station (ISS), as well as time spent on the ISS.

We and others have identified apparent widespread brain gray matter volume (GMv) decreases around the base of the brain and regional GMv increases in the sensorimotor cortices after missions shorter than 6 months (Koppelmans et al. 2016; Roberts et al. 2017; Van Ombergen et al. 2018). Astronauts who completed multimonth missions aboard the ISS showed larger brain changes than those who spent 2 weeks on a Space Shuttle mission (Koppelmans et al. 2016). Ventricular expansion and extracellular fluid shifts (free water measured with diffusion MRI, dMRI) are also greater in ISS compared with Space Shuttle astronauts (Pasternak et al. 2009; Roberts et al. 2017; Asemani et al. 2019; Lee et al. 2019; Riascos et al. 2019). Thus, spaceflight appears to affect brain structure and fluid distribution in a manner that may depend in part on flight duration. However, no studies have examined the impact of spaceflight missions lasting longer than 6 months (Van Ombergen et al. 2018). It therefore remains unknown whether brain changes plateau after some time period in space or if they continue over 1 year of exposure. The design of future missions can be informed by understanding whether brain changes scale parametrically with spaceflight exposure durations of up to 1 year.

Most previous work examining structural brain changes with spaceflight has been limited to one time point before flight and one time point after flight (Koppelmans et al. 2016; Roberts et al. 2017; Lee et al. 2019), which limits the examination of recovery time courses. It is thus unknown whether spaceflight-related brain changes return to baseline levels within days, weeks, months or longer after return to Earth. Characterization of postflight recovery patterns is particularly important given, for instance, the significant pre- to postflight fluid shift changes we have previously reported (Lee et al. 2019).

Specific mechanisms of brain changes with spaceflight are largely unknown. Understanding mechanisms is further complicated by two seemingly conflicting patterns of brain changes with spaceflight—dysfunction and adaptive plasticity. That is, some brain changes appear to be dysfunctional, such as declines in white matter microstructure, where greater pre- to postflight white matter declines in tracts involved in vestibular processes associated with poorer postflight balance (Lee et al. 2019). Other brain changes appear to be adaptive, such as postflight increases in GMv in leg somatosensory cortex (Koppelmans et al. 2016). Long duration spaceflight may result in neuroplastic changes such as axon sprouting, dendritic branching, and changes in glial number and morphology (Zatorre et al. 2012), which could result in measurable structural changes in human sensorimotor brain regions. It is also possible that, in addition to specific sensorimotor structural plasticity and positional shifts of the brain upwards with spaceflight, factors such as sleep loss, radiation, and other spaceflight-related stressors could result in nonspecific brain atrophy or edema.

In fact, there is evidence for both ventricular expansion and extracellular fluid shifts with spaceflight (Roberts et al. 2017; Lee et al. 2019; Roberts and Petersen 2019; Van Ombergen et al. 2019). Ventricular expansion quantifies cerebrospinal fluid (CSF) volume changes within the ventricles (e.g., within the lateral ventricles). Extracellular fluid shifts are calculated using a novel postprocessing technique on dMRI scans. This technique quantifies “free water” (FW), which is defined as water molecules that are not hindered or restricted by their surroundings (Pasternak et al. 2009). FW is found in the ventricles, around the brain parenchyma, and in the extracellular space. FW analysis is therefore a useful tool to investigate cerebral fluid shifts that occur with spaceflight. Multiple studies have found ventricular volume increases with flight (Roberts et al. 2017; Roberts and Petersen 2019; Van Ombergen et al. 2019). Our recent work has identified increased FW at the base of the cerebrum and decreases along the posterior vertex (Lee et al. 2019), suggesting an upward position shift of the brain with spaceflight.

Ventricular volume and FW shifts with spaceflight may be linked to serious functional consequences. One hypothesis of the underlying pathophysiology of spaceflight-associated neuro-ocular syndrome (SANS), a condition affecting up to 50% of astronauts who complete long-duration missions (Mader et al. 2011; Stenger et al. 2017), is that brain fluid and positional shifts slow fluid drainage from the brain. Thus, it is critical to understand the rate at which such fluid and structural changes recover after return to Earth and whether recovery time courses scale with mission duration. Only 2 studies to date have examined recovery of structural brain changes after return from 6-month ISS missions (Van Ombergen et al. 2019; Kramer et al. 2020). These investigators found persisting ventricular volume increases at 7 months and 1 year after flight, respectively, providing compelling preliminary support for the notion that brain fluid distribution changes may recover quite slowly. Thus, further work with larger sample sizes and additional postflight time points is clearly warranted in order to more fully characterize brain recovery after spaceflight.

Of note, aging is also associated with ventricular expansion (Apostolova et al. 2012) and increased FW (Chad et al. 2018). Larger ventricular volume is associated with poorer cognitive function in aging cohorts (Carmichael et al. 2007). However, we suspect that fluid shifts related to spaceflight fundamentally differ from aging processes—instead representing a fluid drainage problem that could have detrimental effects on surrounding tissues. Spaceflight-related fluid shifts may have negative consequences for cognitive function, although characterizing relationships between cognitive function and fluid shifts is beyond the scope of the present work.

Here we compare the effects of 12-month ISS missions (*n* = 2 astronauts) with 6-month missions (*n* = 10 astronauts) on human brain structure and intracranial fluid distribution. We anticipated that changes in ventricular volume, FW, GMv, cortical thickness (CT), and cerebellar volume would be evident in several a priori-selected regions of interest (ROIs) and that changes would scale with flight duration. These regions—lateral ventricles, pre/postcentral gyri, supplementary motor area (SMA), frontal pole, and cerebellum—were selected based on our past work (Koppelmans et al. 2016) and the work of others (Roberts et al. 2017; Van Ombergen et al. 2019) that suggested ventricular expansion and an upward positional shift of the brain with spaceflight, as well as behavioral evidence suggesting that spaceflight may particularly affect sensorimotor control (Mulavara et al. 2010; Cohen et al. 2012; Mulavara et al. 2012). We also tracked these changes out to 6 months after the flights to determine whether postflight recovery toward preflight values takes longer after 12 months than 6 months of spaceflight. Finally, to explore possible predictors of spaceflight-related brain changes, we tested for correlations between slopes of brain change and factors such as flight duration and past flight experience.

## Materials and Methods

### Participants

#### Astronaut Participants

Twelve astronauts participated in this study. Ten of the astronauts completed an ISS mission lasting approximately 6 months, and 2 completed a nearly 12-month-long mission. The two 12-month astronauts provided consent for their data to be presented individually. Astronaut demographics are presented in Table 1.

**Table 1:** Demographics and flight experience for 6-month astronauts^a^

	**6-month astronauts (*n* = 10)** ^**b**^
Sex^c^	9 males; 1 female
Age at baseline scan, mean (SD), years^d^	48 (6)
Number of flight days this mission, mean (SD), days	162 (25)
Naïve or experienced flyer	6 naïve; 4 experienced
Number of previous missions, mean (SD), number of missions	0.7 (1.0)
Number of previous flight days, mean (SD), number of flights	57 (115)
Intermission time^e^, mean (SD), days	2009 (270)
Intermission time^e^, mean (SD), years	5.5 (0.7)

^a^Individual data are not presented here for the 12-month astronauts to protect their privacy. Both of the 12-month astronauts gave permission for their brain data to be depicted in this work as single subject results.

^b^ *n* = 10 in each case, except for intermission time where *n* = 4 because six of the 6-month astronauts were naïve flyers.

^c^One of the 12-month astronauts was male, and the other was female.

^d^Both of the 12-month astronauts fell within 2 standard deviations of the mean age of the 6-month astronauts.

^e^Intermission time is calculated as the time from the previous flight’s landing day to the launch day of the present flight.

#### Control Participants

Brain structure changes throughout the lifespan. Normal, healthy aging is associated with brain volume decreases and ventricular expansion (Fjell and Walhovd 2010). Since spaceflight occurred over 6–12 months, we expected astronauts to exhibit spaceflight-induced brain changes in addition to aging-related brain changes. To characterize typical age-related brain changes, we obtained and analyzed longitudinal sets of neuroimaging data from healthy, ground-based control subjects. We downloaded T1 control data from the Open Access Series of Imaging Studies (OASIS, https://www.oasis-brains.org; Fotenos et al. 2005) for 20 cognitively normal adults (59 ± 7 years). We downloaded dMRI control data from the Alzheimer’s Disease Neuroimaging Initiative (ADNI) database (adni.loni.usc.edu) for 14 cognitively normal adults (67 ± 3 years). See Table 2 for control group demographics.

**Table 2:** Demographics and MRI scans for control groups

	*T*_1_ control group (*n* = 20)	dMRI control group (*n* = 14)
Sex	10 males; 10 females	4 males; 10 females
Years of education, mean (SD), years	16.5 (2.1)	Unknown
Mini-Mental State Exam Score at Baseline, mean (SD), score out of 30	29.5 (1.0)	Unknown
Age at first scan, mean (SD), years	59 (6.7)	67 (2.8)
Number of scans, mean (SD), number^a^	4.7 (1.1)	2
Length of follow-up period, mean (SD), years^b^	7.2 (1.5)	1.4 (0.6)

^a^Number of scans indicates total number of *T*_1_ or dMRI scans completed. Members of the *T*_1_ control group completed a variable number of scans (between 3 and 7 scans). All members of the dMRI control group completed 2 scans.

^b^Length of follow-up period indicates the average time between each person’s first and last scan.

Selection of T1 Control Participants. We selected the OASIS database (specifically, the OASIS-3 dataset) because it includes longitudinal *T*_1_-weighted scans for >600 healthy adult participants over the course of multiple years. These data provide a basis to estimate slopes of expected structural brain change with normal aging. To select 20 individuals for the *T*_1_ control group, we searched the available data for individuals with ≥3 *T*_1_-weighted MRI scans on a 3 T Siemens scanner with no known neurologic disease, cardiovascular condition, or other major health concerns. As most OASIS-3 participants were older than our astronaut sample, we sorted the sample by age. We then selected the youngest 10 males and the youngest 10 females who met our inclusion criteria.

Selection of dMRI Control Participants. We searched the ADNI3 dataset for cognitively normal individuals between 50 and 70 years of age for whom 2 dMRI scans were available. We narrowed results by searching for subjects whose dMRI scans were acquired on a 3 T Siemens scanner using a single-shell sampling scheme. We selected data from all 14 individuals whose dMRI scans met these criteria.

The ADNI3 data were obtained from adni.loni.usc.edu. The ADNI was launched in 2003 as a public-private partnership, led by Principal Investigator Michael W. Weiner, MD. The primary goal of ADNI has been to test whether serial magnetic resonance imaging (MRI), positron emission tomography (PET), other biological markers, and clinical and neuropsychological assessment can be combined to measure the progression of mild cognitive impairment (MCI) and early Alzheimer’s disease (AD). For up-to-date information, see www.adni-info.org.

### Image Acquisition

#### Astronauts

**Figure 1:**
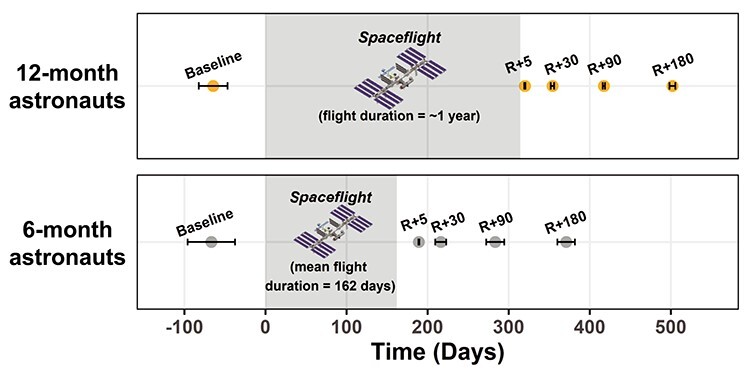
Astronaut Testing Timeline. MRI scan day is shown in relation to flight start and end. Average scanning day ± standard deviation is presented. Day = 0 is the start of flight. R = Return. To protect their privacy, individual data are not presented here for the 12-month astronauts.

We acquired *T*_1_-weighted and dMRI scans at 5 time points: 60 days prior to launch (Baseline) as well as 5, 30, 90, and 180 days after return (R) to Earth (Return+5, Return+30, Return+90, and Return+180, respectively; Fig. 1). One of the 6-month astronauts withdrew from the study before the R + 180 session; thus, we acquired data from nine 6-month astronauts at this time point. All astronaut MRI scans were collected on the same 3 T Siemens Magnetom Verio MRI scanner at University of Texas Medical Branch at Victory Lakes.

The imaging parameters were as follows for the *T*_1_-weighted scans: magnetization-prepared rapid gradient-echo (MPRAGE) sequence, repetition time (TR) = 1900 ms, echo time (TE) = 2.32 ms, flip angle = 9°, field of view (FOV) = 250 × 250 mm, slice thickness = 0.9 mm, 176 slices, matrix = 512 × 512, and voxel size = 0.489 × 0.489 × 0.9 = 0.2152 mm^3^. Diffusion-weighted MRI (dMRI) scans were obtained using a diffusion-weighted 2D single-shot spin-echo prepared echo-planar imaging sequence with the following acquisition parameters: TE = 95 ms, TR = 11 300 ms, flip angle = 90°, FOV = 225 × 225 mm, matrix size = 128 × 128, 40 axial slices of 2 mm slice thickness with zero gap, resulting in a voxel size of 1.95 × 1.95 × 2 mm^3^. Thirty noncollinear gradient directions with diffusion weighting of *b* = 1000 s/mm^2^ were repeatedly sampled 2 times. At the beginning of each sampling stream, we acquired a volume with no diffusion weighting (*b* = 0 s/mm^2^).

#### T1 Control Group

All T1 control data were collected on a 3 T Siemens scanner using standard, high-resolution *T*_1_ acquisition parameters. Further information regarding *T*_1_ image acquisition for the OASIS Longitudinal project can be found at: https://www.oasis-brains.org. These data are publicly available after signing a data use agreement and obtaining approval.

#### dMRI Control Group

All dMRI control data were acquired on a 3 T Siemens scanner (Verio, Prisma, or Skyra). dMRI scans were obtained using diffusion-weighted 2D single-shot spin-echo prepared echo-planar imaging. All acquisitions consisted of a volume with no diffusion weighting (*b* = 0 s/mm^2^) and between 30 and 60 diffusion-weighted volumes at a *b*-value of 1000 s/mm^2^. Acquisition parameters such as TE, TR, number of slices, and voxel size differed across subjects but were consistent within each subject. For further details, see ADNI MRI Scanner Protocols, 2016.

Of note, the *T*_1_ and dMRI control scans were collected on different scanners and had varying acquisition parameters from the astronaut scans. However, parameters were similar between astronauts and controls; for instance, the astronaut dMRI scans were collected with 30 directions, and the control dMRI scans were collected with between 30 and 60 directions (consistent within each subject). Moreover, to minimize scanner effects, our between-group analyses compared the slope of change within subject over time (collected on the same scanner), instead of comparing absolute values of change between groups. Although the different acquisition protocols may still affect the direct comparison between astronauts and controls, the primary purpose of including the control groups was to demonstrate that the observed spaceflight-related brain changes were larger than those observed with normal aging.

### ROI Selection

We measured lateral ventricular volume. Additionally, we extracted GMv and FW for four a priori-selected structural ROIs related to sensorimotor processing: right precentral gyrus, right postcentral gyrus, right SMA, and right frontal pole. The right frontal pole ROI was selected based on peak differences in GMv change from pre- to postflight between ISS and Space Shuttle astronauts in our previous work (Koppelmans et al. 2016). We also examined CT in the right pre- and postcentral gyri, and we tested 4 cerebellar ROIs based on our past work (Bernard and Seidler 2013). No left side cortical ROIs were examined due to an imaging artifact in 1 of the 12-month astronauts.

### GMv Estimation for Pre- and Postcentral Gyri and SMA

*T*_1_ MRI scans were processed with the Computational Anatomy Toolbox (CAT12.6, version 1450; Dahnke et al. 2013; Gaser and Kurth 2017) for Statistical Parametric Mapping Version 12 (SPM12; version 7219; Ashburner et al. 2014) using MatLab R2016a, version 9.0. We used standard CAT12 preprocessing steps (Gaser and Kurth 2017), using all default parameters for longitudinal data, including the new adaptive probability region-growing skull stripping method. All resulting GMv segments were visually inspected and passed acceptable CAT12 quantitative quality control thresholds (i.e., noise, bias, and image quality). Three structural GMv ROIs, right precentral gyrus, right postcentral gyrus, and right SMA and total intracranial volume (TIV), were automatically estimated by CAT12 using the Neuromorphometrics volume-based atlas map (http://Neuromorphometrics.com). GMv ROIs were estimated in native subject space before any spatial registration or normalization (Gaser and Kurth 2017). All GMv results were corrected for head size using TIV at the baseline time point with the formula: (ROI volume/TIV at baseline scan)*100.

### Ventricular Volume Estimation

Ventricular volume was also estimated using CAT12 with identical methods to the above GMv ROIs. CAT12 automatically estimated lateral ventricular volume in native space using the Neuromorphometrics volume-based atlas map. To account for differences in head size, ventricular volume is presented as percent change from baseline scan.

### CT Estimation for Pre- and Postcentral Gyri

The CAT12 preprocessing pipeline also includes extraction of surface-based morphometry metrics. These surface estimations use a fully automated method that employs a projection-based thickness algorithm to measure CT and reconstruct the central cortical surface (Gaser and Kurth 2017). CT ROI measures were estimated in native subject space before any spatial registration or normalization (Gaser and Kurth 2017) and were based on structures defined by Desikan-Killiany gyral-based atlas (Desikan et al. 2006). CT results were not corrected for TIV, as head size does not significantly affect CT (Gaser and Kurth 2017). Prior to analysis, the quality of the extraction of cortical surface data was examined visually for each scan using the “Display Surfaces” tool within CAT12.

### GMv Estimation for Frontal Pole ROI

We extracted the coordinates of peak difference in pre- to postflight change (on the right side of the brain) between 13 Space Shuttle astronauts (~2 week flight) and 14 ISS astronauts (~6 month flight) from our analysis of retrospective astronaut MRI scans: right frontal pole (Montreal Neurological Institute (MNI) coordinates = 32, 37, −16; Koppelmans et al. 2016). In this region, ISS crewmembers showed greater decreases in GMv from pre- to postflight compared with changes evident in Space Shuttle crewmembers.

While the structural ROI volumes (right pre- and postcentral gyri and SMA) were estimated in native space, the right frontal pole ROI was estimated from the unsmoothed, modulated GM images returned by CAT12. Briefly, this additional CAT12 processing included the following: for each subject, all scans were bias-corrected between time points and registered to the mean image of all time points using an inverse-consistent realignment. The mean image was segmented into gray matter, white matter, and cerebrospinal fluid. Spatial normalization parameters were estimated for the mean image using high-dimensional Dartel registration. Images for each time point were then segmented, normalized to template space using the parameters from the mean image, and modulated. To create the ROI, we used the Wake Forest University PickAtlas toolbox (Maldjian et al. 2003) to make a spherical mask with a 5 mm radius and resliced the mask to match the voxel size of a Dartel GM segmentation (1.5 × 1.5 × 1.5 mm^3^). To calculate the volume inside the ROI, we used the get_totals.m script by Ged Ridgway (http://www0.cs.ucl.ac.uk/staff/g.ridgway/vbm/get_totals.m), setting images = subjects’ unsmoothed GM segments, mask = each respective spherical ROI mask, and threshold = 0. To account for head size, frontal pole GMv results were corrected using TIV at the baseline time point with the formula: (ROI volume/TIV at baseline scan)*100.

### Estimation of Cerebellar Volumes

To estimate cerebellar lobular volumes, we used the CEREbellum Segmentation (CERES) pipeline (Romero et al. 2017), which employs a patch-based multiatlas segmentation tool to automatically segment and parcellate the cerebellum into 26 structures. Ultimately, CERES calculates volumes of these structures in native space. CERES processing has been described in detail elsewhere (Romero et al. 2017) and has been shown to perform better than semi-automatic or manual segmentation methods (Romero et al. 2017). After processing of cerebellar data with CERES, the resulting tissue segmentation was individually checked against each subject’s filtered, normalized MNI registered image for goodness of fit and anomalies.

We present cerebellar total volumes for each region rather than GMv to avoid any inaccuracies due to low contrast differences between cerebellar gray matter and white matter. To minimize the number of comparisons, we summed individual lobule volumes to create 4 ROIs based on our past work (Bernard and Seidler 2013): anterior cerebellum, posterior cerebellum, left crus I, and right crus I. These ROIs were based on principal components derived from our previous analysis of cerebellar data from 23 young adults (Bernard and Seidler 2013) (Supplementary Table 1). We summed these values for each of the 4 ROIs, as defined in Supplementary Table 1. To account for differences in head size, all cerebellar volumes are presented as percent change from the baseline scan.

### dMRI and FW Processing

We used the FMRIB Software Library (FSL) version 6.0.1, MATLAB R2018b, Advanced Normalization Tools (ANTs 2.1.0; Avants et al. 2010; Avants et al. 2011), and custom-written FW imaging algorithms (Pasternak et al. 2009) for analysis of dMRI images. All raw dMRI images were visually inspected for volumes with subject motion or scan artifacts. We then used standard dMRI preprocessing. Rician filter was applied to the dMRI data to remove random noise (Manjón et al. 2013). FSL’s eddy tool was used to correct for eddy current-induced distortions, subject movement, and accompanying b-vector rotation. All dMRI volumes were registered to *b* = 0 volume. We generated subject movement plots reflecting root mean square (RMS) deviations of the registration parameters. Any volume that was displaced more than 1 mm relative to the previous volume was deemed an outlier and was removed from the 4D eddy-corrected data and the b-value and b-vector matrices. The resulting images were then skull-stripped using FSL’s brain extraction tool.

FW images were produced for each individual and each time point, utilizing an in-house developed algorithm that uses a bitensor model (Pasternak et al. 2009). These FW images represent the fractional volume of FW in a voxel, which is the proportion of water molecules that are not restricted or hindered by their surroundings. This algorithm also generates fractional anisotropy (FA) images reflecting the preferred directionality of water diffusion for each voxel. We used a three-step process to normalize FW images to MNI152 standard space. First, adapting the processing pipeline described by Schwarz et al. (2014), we created subject-specific FA template in a way that was unbiased between the input images of any specific time point account for the longitudinal nature of the data. We constructed subject-specific templates using the ANTs function antsMultivariateTemplateConstruction.sh. Next, we warped the individual FA templates to MNI152 standard space using ANTs’ SyN algorithm.

Finally, we combined the linear transformations and nonlinear warp parameters from the individu*al FA* image to the subject specific FA template and the MNI152 common space into one flow field. For each subject and each session, the corresponding flow field was applied to the FW image to transform it to MNI152 standard space. FW images were resliced to 1 mm^3^. Mean FW values within each ROI were extracted from the nonsmoothed FW images. To match the GMv structural ROIs, we obtained each ROI from the Neuromorphometrics atlas (http://Neuromorphometrics.com) provided in SPM12 (Ashburner et al. 2014) and resliced to MNI152 standard space. The 5 mm spherical custom right frontal pole ROI was also created in the identical space.

### Creation of Cine Clips (Supplementary Movies 1 and 2)

Similar to the procedure used by Roberts et al. (Roberts et al. 2017), we rigidly coregistered the raw pre- and postflight image for each of the 12-month astronauts in SPM and created short cine clips showing selected flight-related brain changes (Supplementary Movies 1 and 2). These videos are meant to be illustrative, in order to help demonstrate several of our primary findings.

### Statistics

#### Visual Comparisons

**Figure 2:**
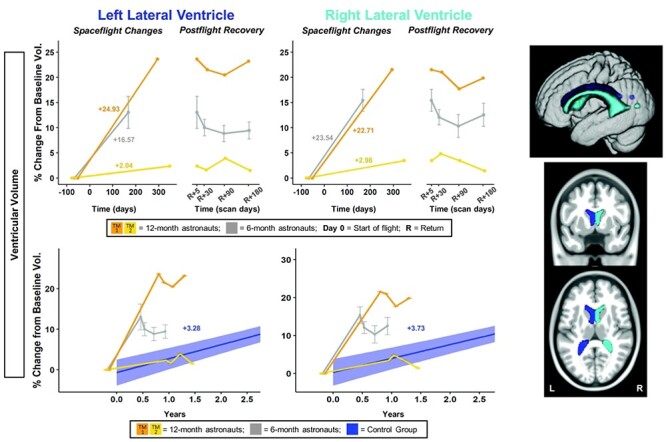
Ventricular volume changes with spaceflight and comparison with aging. Twelve-month astronaut data are shown in orange (TM-1) and yellow (TM-2). Six-month astronaut data are shown in gray. Volume changes are expressed as a percent change from baseline scan. Top: Numeric values on the left “Spaceflight Changes” panels indicate slope of change in units of volume (% of baseline ventricular volume). For the 6-month group, slopes are the group median slope. Error bars indicate standard error. Structural ROIs are overlaid onto slices and a rendered template brain in standard space. Bottom: The average brain change over time for the control participants is indicated by the blue line with blue 95% confidence interval. The control group median slope is indicated in blue text. Error bars indicate standard error. The dMRI control scans were collected over an average of 1.4 ± 0.6 years and thus entire trajectory of change for the dMRI control data is depicted in the figure. ROI Image: Structural ROIs are overlaid onto slices and a rendered template brain in standard space.

**Figure 3:**
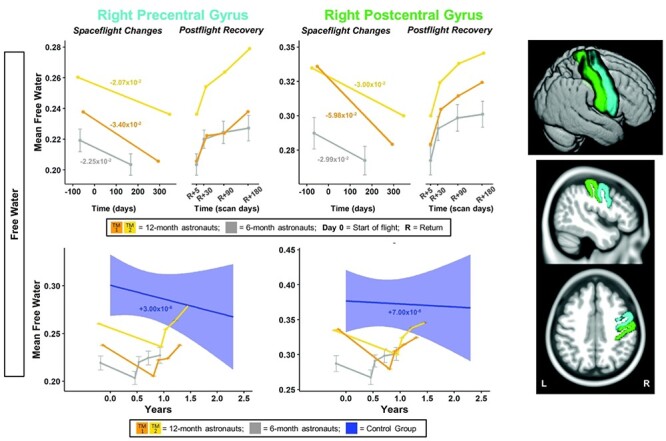
Pre-/postcentral gyri FW changes with spaceflight and aging. Twelve-month astronaut data are shown in orange (TM-1) and yellow (TM-2). Six-month astronaut data are shown in gray. Control data are shown in blue. FW is expressed as mean FW fraction change per year. Top: Numeric values in the left “Spaceflight Changes” panels indicate slope of change in units of mean FW fraction per year. For the 6-month group, these slopes are the group median slope. Error bars indicate standard error. Bottom: The average FW change over time for the control participants is indicated by the blue line with blue 95% confidence interval. The control group median slope is indicated in blue text. Error bars indicate standard error. The dMRI control scans were collected over an average of 1.4 ± 0.6 years and thus entire trajectory of change for the dMRI control data is depicted in the figure. ROI Image: Structural ROIs are overlaid onto slices and a rendered template brain in standard space.

**Figure 4:**
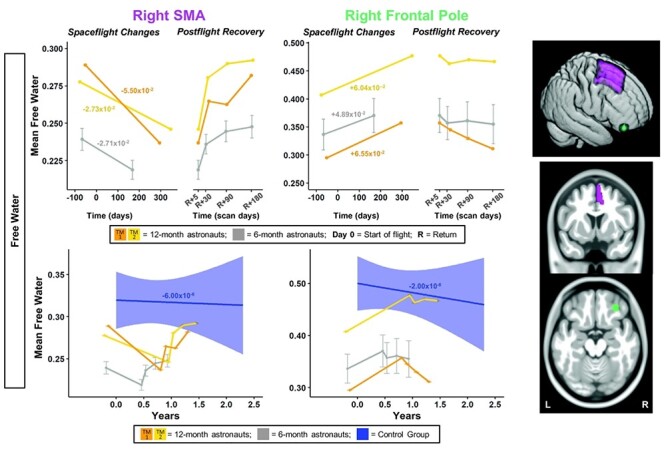
SMA and frontal pole FW changes with spaceflight and aging. Twelve-month astronaut data are shown in orange (TM-1) and yellow (TM-2). Six-month astronaut data are shown in gray. Control data are shown in blue. FW is expressed as mean FW fraction change per year. Top: Numeric values in the left “Spaceflight Changes” panels indicate slope of change in units of mean FW fraction per year. For the 6-month group, these slopes are the group median slope. Error bars indicate standard error. Bottom: The average FW change over time for the control participants is indicated by the blue line with blue 95% confidence interval. The control group median slope is indicated in blue text. Error bars indicate standard error. While the *T*_1_ control scans were collected over an average of 7.2 ± 1.5 years, the *x*-axis here only continues out to 2.75 years in order to provide better visual comparison with the astronaut data. ROI Image: Structural ROIs are overlaid onto slices and a rendered template brain in standard space.

**Figure 5:**
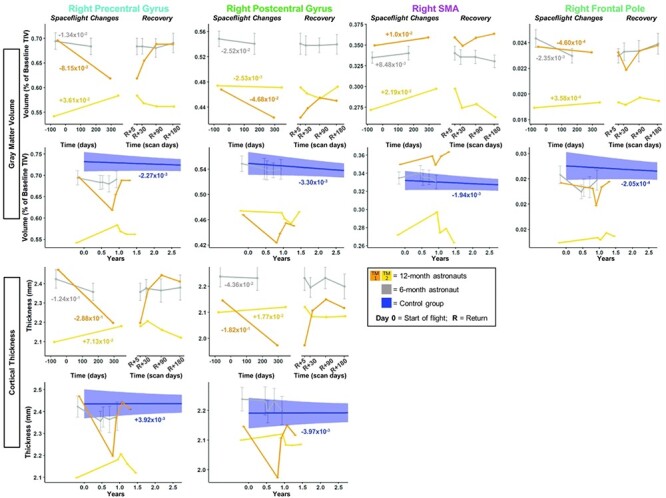
Pre-/postcentral gyri, SMA, and frontal pole GMv and CT changes with spaceflight and aging. Twelve-month astronaut data are shown in orange (TM-1) and yellow (TM-2). Six-month astronaut data are shown in gray. Control data are shown in blue. GMv changes are expressed as a percentage of preflight total intracranial volume (TIV) at baseline scan. CT is expressed as thickness (mm) change per year. Spaceflight Graphs: Numeric values in the left “Spaceflight Changes” panels indicate slope of change in units of volume (% of baseline TIV) or thickness (mm) per year. For the 6-month group, these slopes are the group median slope. Error bars indicate standard error. Control Group Graphs: The average FW change over time for the control participants is indicated by the blue line with blue 95% confidence interval. The control group median slope is indicated in blue text. Error bars indicate standard error. While the *T*_1_ control scans were collected over an average of 7.2 ± 1.5 years, the *x*-axis here only continues out to 2.75 years in order to provide better visual comparison with the astronaut data.

**Figure 6:**
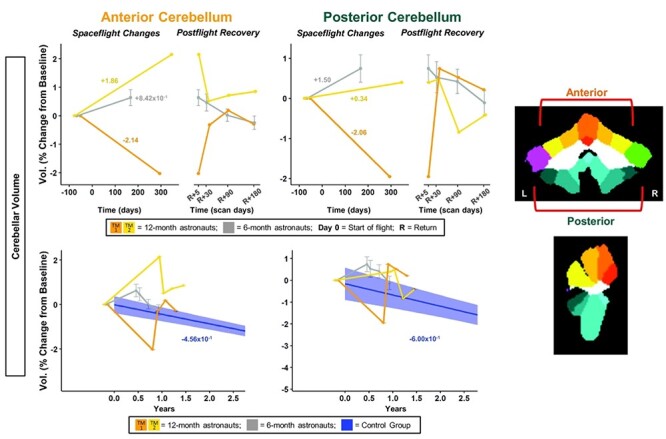
Cerebellar volume changes with spaceflight and aging. Twelve-month astronaut data are shown in orange (TM-1) and yellow (TM-2). Six-month astronaut data are shown in gray. Volume changes are expressed as a percent change from baseline scan. Top: Numeric values on the left “Spaceflight Changes” panels indicate slope of change in units of % of baseline volume per year. For the 6-month group, these slopes are the group median slope. Error bars indicate standard error. Bottom: The average brain change over time for the control participants is indicated by the blue line with blue 95% confidence interval. The control group median slope is indicated in blue text. Error bars indicate standard error. While the T1 control scans were collected over an average of 7.2 ± 1.5 years, the *x*-axis here only continues out to 2.75 years in order to provide better visual comparison with the astronaut data. ROI Image: ROIs are indicated on template in CERES space. Warm-colored regions indicate those included in anterior cerebellum ROI. Cool-colored regions indicate those included in posterior cerebellum ROI.

Given the small but unique sample size for the 12-month astronauts, no statistical tests were performed to compare ROI values between the 12- and 6-month astronauts. Instead, we present qualitative comparisons of brain changes (Figs 2–6).

#### Slopes of Brain Change

For the astronauts and controls, we calculated the slopes of brain changes for each ROI in units of change per year. For the astronauts, we included the ROI values at baseline and Return+5 days in this slope calculation to describe brain changes with flight. For the control groups, for each subject’s slope, we included all available time points (ranging from 3 to 7 for the *T*_1_ group and 2 time points for the dMRI group). Given the sample size and nonnormal distributions, we ran nonparametric unpaired two-samples Wilcoxon rank sum tests to compare slopes of brain change with flight for the 6-month astronauts to slopes of brain change with normal aging (Figs 2–6; Supplementary Table 2). We present slopes for the 12-month astronauts in Supplementary Table 2 for qualitative comparisons.

#### Postflight Recovery

We examined the recovery trajectory for the 6-month astronauts using a linear mixed model with restricted maximum likelihood estimation for the 4 postflight time points (Supplementary Table 3). The model included a random intercept for subject (to allow for different baseline values for each person) and the fixed effect of time. We interpreted a significant fixed effect of time as the occurrence of postflight recovery for the 6-month astronauts (Figs 2–6). In Supplementary Table 3, we also present the percentage of recovery achieved by 6 months after flight; we calculated this as: [(Return+5 days value–Return+180 days value)/(Return+5 days value)*100%] on the values of percentage change from baseline.

#### Exploratory Correlations

We tested nonparametric Spearman correlations between pre- to postflight slope of brain change and the following variables: 1) intermission time (i.e., time between end of last mission and present flight launch); 2) number of previous missions; 3) past number of flight days; and 4) current number of flight days. We also tested relationships between preflight ventricular volume and each of the above variables.

## Results

### Astronaut Slopes of Brain Change

#### Fluid Shifts

Twelve months in space generally resulted in larger fluid shifts than 6 months in space (Figs 2–4, Supplementary Table 2). We found substantial ventricular enlargement with flight for 1 of the 12-month astronauts (TM-1; shown in Fig. 2 in orange) and for the ten 6-month astronauts. TM-1 had volumetric increases of 25% and 23% (i.e., ~ 1–1.3 mL in each case) within the left and right lateral ventricles, respectively. See Supplementary Movie 1 for a video clip illustrating TM-1’s ventricular enlargement. For the 6-month group, the median slope of change in ventricle volume was 17 ± 12% and 24 ± 6% for the left and right, respectively. The second 12-month astronaut (TM-2; shown in Fig. 2 in yellow) exhibited small increases in ventricular volume of 2% and 3% (i.e., 0.4–0.6 mL), but this individual exhibited larger ventricles to start with, perhaps as a result of prior spaceflights.

For the right frontal pole, the pre- to postflight slope of increase in FW was steeper for both of the 12-month astronauts compared with the 6-month astronauts (Fig. 4). For FW within the right pre-/postcentral gyri and SMA, TM-2 (Figs 3 and 4 yellow) showed similar decreases compared with the 6-month group, while TM-1 (Figs 3 and 4 orange) showed greater decreases in FW in these regions compared with the 6-month group.

#### GMv and CT Changes

The pre- to postflight GMv and CT slope was steeper for both of the 12-month astronauts compared with the 6-month astronauts for several measures: right precentral gyrus GMv/CT and right SMA GMv (Fig. 5; Supplementary Table 2). For postcentral gyrus GMv and CT, only TM-1 showed a steeper slope of change with flight (Fig. 5 orange). Both 12-month astronauts exhibited *smaller* changes in GMv within the frontal pole compared with the 6-month astronauts (Fig. 5; Supplementary Table 2).

The 6-month astronauts showed decreases with flight in GMv and CT for each of these regions, except for the SMA, where TM-1 and the 6-month group showed increases with flight. For the right pre/postcentral gyri, TM-2 (Fig. 5 yellow) showed a different pattern of GMv and CT change than TM-1 and the 6-month group. It is unclear why TM-2 experienced these opposite directions of change. We elaborate on potential mechanisms in Discussion.

#### Evidence for Upwards Shift of the Brain with Spaceflight

The pre-/postcentral gyri and SMA exhibited FW decreases paired with GMv and CT increases for TM-2 (Figs 2–5 yellow). This could represent an upward shift of the brain from pre- to postflight (Koppelmans et al. 2016; Roberts et al. 2017). See Supplementary Movie 2 for further support of this hypothesis; this clip depicts vertex compression for TM-2.

#### Cerebellar Volume Changes

For the anterior cerebellum, the 12-month astronauts had steeper slopes of volume change compared with the 6-month group (Fig. 6; Supplementary Table 2). TM-2 (Fig. 6 yellow) showed a steeper increase in volume compared with the 6-month astronauts, while TM-1 (Fig. 6 orange) showed a steeper decrease. For the posterior cerebellum, TM-2 showed a smaller increase in volume compared with the 6-month astronauts, while TM-1 again showed a steep decrease. For right crus I, the 12-month astronauts both showed greater decreases in volume compared with the 6-month astronauts (Supplementary Fig. 1; Supplementary Table 2). For left crus I, all astronauts showed little change with flight (Supplementary Fig. 1; Supplementary Table 2).

### Comparison of Brain Change Slopes with Control Group

We evaluated whether these brain changes described above were greater than would be expected with healthy aging over a comparable timeframe on Earth. It should be noted that the controls were older than the astronauts (Table 2), and would therefore be expected to exhibit steeper declines over time.

#### Fluid Shifts

The 6-month astronauts and TM-1 (Fig. 2 orange) showed greater ventricular expansion than the control group (Supplementary Table 2); thus, these changes were larger than what would be expected with normal aging. The 6-month astronauts showed ventricular expansion at a rate 5–6 times that of the controls, and TM-1 showed ventricular expansion at a rate 6-8 times that of controls (Supplementary Table 2). TM-2 (Fig. 2 yellow) showed ventricular expansion similar to that of normally aging individuals (Supplementary Table 2).

Compared with the controls, the 6-month astronauts showed a larger slope of change for pre−/postcentral gyrus FW (Fig. 3; Supplementary Table 2). Slopes of change in pre−/postcentral gyri FW for the 12-month astronauts were between ~4000 and >11 000 times greater in magnitude than slopes of change with normal aging (Fig. 3; Supplementary Table 2).

FW slopes of change within the SMA for the 6-month astronauts were significantly greater than those of the control subjects (Fig. 4; Supplementary Table 2). The 12-month astronauts exhibited a steeper slope of change than the control group for SMA FW; 12-month slopes were between ~4000 and >9000 times steeper than those of the controls (Fig. 4; Supplementary Table 2).

The 6-month astronauts exhibited a steeper slope of increase than the controls for frontal pole FW (Fig. 4; Supplementary Table 2). The slopes of frontal pole FW change for the 12-month astronauts were ~30 000 times steeper than the controls (Fig. 4; Supplementary Table 2).

#### GMv and CT Changes

Compared with the controls, the 6-month astronauts showed a larger slope of change for precentral gyrus CT and for postcentral gyrus GMv (Fig. 5; Supplementary Table 2). With the exception of right postcentral gyrus GMv for TM-2, slopes of change in pre−/postcentral gyri GMv and CT for the 12-month astronauts were between 5 and 74 times greater in magnitude than slopes of change with normal aging (Fig. 5; Supplementary Table 2).

Slopes of change within SMA for GMv for the 6-month astronauts were significantly greater than those of the control subjects (Fig. 5; Supplementary Table 2). 12-month slopes were between 5 and 11 times steeper than those of the controls (Fig. 5; Supplementary Table 2).

For the frontal pole, while the 12-month astronauts showed numerically greater slopes of change for GMv than the controls (~2 times greater), there was no significant difference in GMv slopes between the 6-month astronauts and the controls (Fig. 5; Supplementary Table 2). Thus, frontal pole GMv changes observed following 6 months of spaceflight were no different than would be expected with 6 months of normal aging on Earth.

#### Cerebellar Volume Changes

The increases within the anterior and posterior cerebellum for the 6-month group were significantly different from the control group (Fig. 6; Supplementary Table 2). For the anterior cerebellum, both of the 12-month astronauts showed steeper slopes than the controls, although TM-2 (Fig. 6 yellow) exhibited a 4 times greater increase in cerebellar volume with flight, while TM-1 (Fig. 6 orange) exhibited a 5 times greater decrease. For the posterior cerebellum, TM-1 (Fig. 6 orange) showed a 3 times steeper decrease compared with controls, while TM-2 (Fig. 6 yellow) showed increased cerebellar volume but with a shallower slope than the controls. For right but not left crus I, the 6-month astronauts had significantly different slopes of change compared with the controls (Supplementary Fig. 1; Supplementary Table 2). The 12-month astronauts exhibited 3-6 times steeper decreases in left and right crus I volumes compared with the controls, with the exception of TM-1, who showed a smaller volume decrease in left crus I compared with the controls (Supplementary Fig. 1; Supplementary Table 2).

### Postflight Changes over Time

In general, most astronauts showed partial or complete return to preflight levels by 6 months postflight. However, there was heterogeneity in these recovery patterns, with several astronauts showing continuing change rather than recovery (see Supplementary Table 3).

#### Fluid Shifts

Ventricular volume only partially returned to preflight levels by 6 months postflight (Fig. 2; Supplementary Table 3). The 6-month astronauts did not exhibit a significant recovery pattern (Supplementary Table 3). A subset of the 6-month astronauts (*n* = 4 for left and *n* = 5 for right side) showed 64% and 55% recovery by 6 months postflight, while the other 6-month astronauts (*n* = 5 for left and *n* = 4 for right side) showed no return toward preflight levels (i.e., continued increases in ventricular volume between Return+5 days and 6 months postflight; Supplementary Table 3). Similarly, left and right ventricular volumes at 6 months recovered by only 2% and 8% for TM-1 (Fig. 2 orange; Supplementary Table 3). TM-2 (Fig. 2 orange), who showed small ventricular volume increases with flight, had recovery of 37% and 59% by 6 months postflight.

The 6-month astronauts showed a significant return toward preflight levels in the 6 months postflight for pre- and postcentral gyri FW (Fig. 3; Supplementary Table 3). The 12-month astronauts exhibited pre- to postflight change in pre/postcentral gyri FW followed by recovery of >100% at 6 months postflight (Fig. 3; Supplementary Table 3).

The 6-month astronauts showed significant return toward preflight levels for SMA FW (Fig. 4; Supplementary Table 3). Both 12-month astronauts visually showed a recovery pattern for FW, with recovery between 87% and >100% at 6 months (Fig. 4; Supplementary Table 3).

The 6-month astronauts showed significant return toward preflight levels for frontal pole FW (Fig. 4; Supplementary Table 3). Both of the 12-month astronauts exhibited partial recovery in frontal pole FW, of 15% and 74% (Fig. 4; Supplementary Table 3).

#### GMv and CT Changes

The 6-month astronauts did not show a significant return toward preflight levels in the 6 months postflight for pre-/postcentral gyri GMv or CT (Fig. 5; Supplementary Table 3). However, the 12-month astronauts exhibited pre- to postflight change in these measures followed by recovery of between 43% and >100% at 6 months for these pre-/postcentral gyri measures (Fig. 5; Supplementary Table 3).

The 6-month astronauts showed significant return toward preflight levels for SMA GMv (Fig. 5; Supplementary Table 3). For SMA GMv, TM-2 (Fig. 5 yellow) showed >100% recovery by 6 months, while TM-1 (Fig. 5 orange) showed partial recovery by Return+30 days, but then exhibited continuing GMv increases between Return+30 days and Return+180 days, instead of recovery.

The 6-month astronauts showed significant return toward preflight levels for frontal pole GMv (Fig. 5; Supplementary Table 3). For GMv, TM-1 showed >100% recovery, while TM-2 showed a 0.59% continued increase from Return+5 to Return+180 days (Fig. 5; Supplementary Table 3).

#### Cerebellar Volume Changes

The 6-month astronauts showed significant return toward preflight levels for anterior and posterior cerebellar volumes (Fig. 6; Supplementary Table 3), but not for left or right crus I volumes (Supplementary Fig. 1; Supplementary Table 3). Visually, both 12-month astronauts showed a recovery pattern for all cerebellar volumes, with the exception of left crus I (Fig. 6; Supplementary Fig. 1). TM-2 (Supplementary Fig. 1 yellow) showed a large increase in left crus I volume at Return+30 days followed by a return toward baseline volume values; the reasons underlying this spike at Return+30 days are unclear. TM-1 (Supplementary Fig. 1 orange) showed large continuing increases from Return+5 days to 6 months postflight.

### Correlations Between Slope of Brain Changes and Flight Experience

#### Intermission Time

The longer the period between a previous flight and the current flight, the greater the increase in left and right ventricular volume during the current mission (Supplementary Fig. 2; Supplementary Table 4). Additionally, a longer time from previous flight was associated with a smaller decrease or greater increase in left crus I volume (Supplementary Fig. 2; Supplementary Table 4).

#### Number of Previous Missions

More previous missions was associated with smaller increases in right ventricular volume, with larger increases in frontal pole FW, and with smaller decreases or larger increases in frontal pole GMv (Supplementary Fig. 3; Supplementary Table 4).

#### Past Number of Flight Days

A greater number of previous flight days was associated with larger increases in frontal pole FW and with smaller decreases or larger increases in frontal pole GMv (Supplementary Fig. 3; Supplementary Table 4).

#### Current Number of Flight Days

A greater number of flight days in the current mission associated with larger increases in frontal pole FW (Supplementary Fig. 3; Supplementary Table 4).

### Correlations between Preflight Ventricular Volume and Flight Experience

Less time between missions correlated with larger left and right ventricular volumes at baseline (Supplementary Fig. 4; Supplementary Table 5). More previous missions and more past flight days correlated with larger baseline right ventricular volume (Supplementary Fig. 4; Supplementary Table 5). These baseline differences did not relate to age; age did not correlate with greater preflight ventricular volume (Supplementary Table 5).

## Discussion

Twelve months in space generally resulted in larger brain changes than 6 months, particularly for fluid shifts. Most measures returned to preflight levels by 6 months after the mission, with the exception of ventricular volume, which showed only partial recovery by 6 months after flight.

We identified GMv and CT increases in pre- and postcentral gyri and SMA for 1 of the 12-month astronauts (TM-2) and the 6-month astronaut group. This could reflect CSF redistribution (Koppelmans et al. 2016; Roberts et al. 2017) or structural neuroplasticity in response to the altered vestibular and reduced somatosensory inputs during spaceflight. Sensorimotor novelty and practice have been associated with positive plasticity and neuroprotection (Latchney et al. 2014; Sale et al. 2014). Moreover, oyster toadfish (a model system for studying spaceflight-related vestibular changes) showed a threefold increase in sensitivity of utricular afferents during spaceflight, providing a potential trigger for cortical plasticity (Boyle et al. 2001). Thus, long-duration spaceflight could induce neuroplastic effects.

For both the 12- and 6-month astronauts, we found flight-related FW decreases paired with GMv increases in SMA. For 1 of the 12-month astronauts (TM-2), we found FW decreases paired with GMv and CT increases in pre/postcentral gyri. Additionally, all astronauts showed postflight increases in frontal pole FW. These findings replicate previous reports of an upward shift of the brain within the skull from pre- to postflight (Koppelmans et al. 2016; Roberts et al. 2017; Lee et al. 2019), including widespread FW increases at the base of the brain and FW decreases near the vertex (Lee et al. 2019), as well as compression of superior gray matter (Koppelmans et al. 2016; Roberts et al. 2017). The decreases in sensorimotor cortex FW identified here may thus result from compression of the brain at the vertex. The concomitant GMv increases in these superior sensorimotor regions could stem from upward brain shift and/or could reflect adaptive neuroplastic processes. The increased frontal pole FW could represent increased fluid near the base of the brain.

We found opposite directions of change for pre- and postcentral gyri GMv and CT for the two 12-month astronauts. Differences between the 2 astronauts such as sex, age, or previous flight exposure could play a role. Further work with additional 12-month astronauts is needed to better understand the underlying causes of individual differences in the responses to spaceflight.

In general, the astronauts showed a progressive recovery pattern that was approximately complete by 6 months after return for all examined brain regions, except for the ventricles. This suggests that many brain structural changes with spaceflight are reversible after return, at least for flights up to 1 year. The ability to examine recovery here represents a novel contribution to the spaceflight neuroscience literature. The majority of past work collecting MRI scans with spaceflight has included only one preflight and one postflight time point (Koppelmans et al. 2016; Roberts et al. 2017; Lee et al. 2019), precluding examination of recovery time courses. For instance, our past work identified widespread FW increases in the frontal, temporal, and occipital lobes and FW decreases at the posterior vertex following spaceflight (Lee et al. 2019). In this previous work, we analyzed one postflight MRI scan collected at an average of 4–11 days after landing. Although these data provide clear evidence for fluid shifts with spaceflight, collecting only one postflight time point does not allow for examination of how quickly after return to Earth these changes return to baseline levels. In the present work, by collecting 4 postflight time points out to 6 months, we identified that the majority of structural brain changes do indeed return to baseline levels by 6 months for both 6- and 12-month astronauts. These data also provide clear evidence that ventricular volume increases with 6–12 months in space do not return to preflight levels by 6 months after flight.

The precise mechanism underlying ventricular expansion with spaceflight and lack of recovery remains unclear. The apparent upwards shift of the brain with flight could lead to compression of the superior sagittal sinus and arachnoid granulations; these structures are primarily responsible for CSF drainage from the brain, so compression at these regions could slow CSF resorption (Sakka et al. 2011). Ventricular expansion is also hypothesized to reflect compensation for altered cerebrospinal fluid hydrodynamics during spaceflight. Roberts and colleagues have termed these spaceflight-induced ventricular volume increases “hydrocephalus associated with long-term spaceflight” (i.e., HALS; Roberts and Petersen 2019). Others have suggested that spaceflight-associated ventricular volume increases are indicative of normal pressure hydrocephalus and glymphatic system dysfunction (Kramer et al. 2020). However, the precise mechanisms for ventricular expansion remain unclear, and it remains unclear why such mechanisms would require many months after return to Earth to return to preflight values.

These ventricular changes and the incomplete recovery that we observed are of concern for future missions, in light of recent work by Van Ombergen and colleagues showing an association between ventricular volume and visual acuity changes with spaceflight (Van Ombergen et al. 2019). Further, it has been hypothesized that brain fluid shifts and ventricular expansion may contribute to the pathophysiology of SANS, which affects up to 50% of long-duration astronauts and poses significant health concerns (Mader et al. 2011; Stenger et al. 2017). This ventricular expansion during longer duration missions could pose risks, including ocular problems such as SANS (Lee et al. 2018) as well as potential interference with cerebrospinal fluid waste drainage to the glymphatic system (Ringstad et al. 2018). It is not clear why ventricular changes fail to fully recover by 6 months postflight. However, it is interesting that ocular changes also appear to require a long time for recovery, with one study finding elevated intracranial pressure up to 19 months after spaceflight (Mader et al. 2011)—suggesting a relationship between lasting fluid shifts and ocular impairments.

Interestingly, a longer time from previous flight was strongly associated with a greater increase in ventricular volume in our study. This suggests that those who had less time between flights may not have recovered fully; therefore, these astronauts began their current flight with elevated ventricular volumes and thus did not exhibit inflight increases due to physiological or structural limits. This is further supported by our present finding that those with less time between missions, more previous missions, and more past flight days had greater preflight ventricular volumes (and that these larger preflight ventricular volumes did not correlate with older age). It is also worth noting that the 12-month astronaut (TM-2) who did not exhibit large flight-related increases in ventricular volume presented with visibly large ventricles at baseline, again supporting the notion of inadequate recovery time between subsequent flights. That is, the time since this astronaut’s previous flight could have been insufficient for full recovery and precluded further room for ventricular expansion over the 12-month mission.

We identified ventricular volume increases of over 20% in some astronauts, without complete return to preflight levels even 6 months postflight. One of the 12-month astronauts (TM-1) had ventricular volume increases that were 6–8% larger than what would be expected with normal aging. Of note, the control group used was older than the astronaut cohort. As ventricular volume expansion is more pronounced in later life (Takao et al. 2012), a younger control group likely would have exhibited even less ventricular volume increase over time, making differences between the astronauts and controls even more pronounced. We identified even greater disparity between the astronaut and the control subjects for other measures, such as precentral gyrus FW, where the 12-month astronauts incurred changes that were >11 000 times greater than those of the control group. This suggests that most of the structural brain changes we have identified are indeed due to spaceflight; they are larger than what is expected with normal aging and may represent either dysfunction or adaptive processes related to spaceflight.

It is difficult to determine specific functional implications of these structural brain changes with spaceflight. Spaceflight results in postflight impairments to behaviors such as postural control (Layne et al. 2001; Cohen et al. 2012; Wood et al. 2015) and locomotion (Bloomberg and Mulavara 2003; Miller et al. 2010; Mulavara et al. 2010). However, there is also evidence of in-flight sensorimotor adaptations such as sensory reweighting (i.e., down-weighting of sensory information that is irrelevant for motor control in microgravity; Lowrey et al. 2014). It thus follows that spaceflight-related brain changes could represent adaptations to the spaceflight environment and/or dysfunctional neural changes. Here we do not correlate behavioral data with the observed brain changes, so we are unable to fully characterize such relationships. For instance, it could be that the observed sensorimotor GMv increases represent neuroplastic adaptations in sensorimotor processing. However, these GMv increases could also be a result of the physical upwards shift of the brain and thus result solely from mechanical compression of the top of the brain. Further work is needed to examine associations between brain changes and behavior to fully understand the functional impacts of these structural changes.

The limited brain—behavioral evidence to date suggests both positive and negative effects of structural brain changes with flight. For instance, we found increased GMv in leg somatosensory cortex with spaceflight (Koppelmans et al. 2016). In our parallel head-down-tilt bed rest work, GMv increases within the same region were associated with smaller decrements in standing balance performance from pre- to postbed rest (Koppelmans et al. 2017). Together, these findings are suggestive of adaptive plasticity to altered sensory inputs in the spaceflight environment. On the other hand, we also found that astronauts who showed the greatest postflight disruptions in white matter structural connectivity in the superior longitudinal fasciculus also showed the greatest postflight balance declines (Lee et al. 2019), supporting that some brain changes with spaceflight are maladaptive.

Past work using electroencephalography (EEG) also provides some insight into potential functional brain changes with spaceflight. One study examined brain oscillations in astronauts before, during, and after spaceflight. Primary motor cortex, as well as vestibular and cerebellar brain areas, showed reduced alpha power during spaceflight. As the alpha rhythm (8–12 Hz) is considered to be an indicator of sensory input inhibition (Pfurtscheller et al. 1996), this reduction is suggestive of greater sensory disinhibition when integrating conflicting visual, vestibular, and proprioceptive inputs during flight. Such sensory reweighting could reflect increased reliance on somatosensory inputs for stabilizing posture while free-floating in microgravity (Cebolla et al. 2016). Other work has similarly shown reductions in alpha band activity in-flight; for instance, 1 study found that visual evoked potentials were suppressed and occipital brain areas exhibited reduced alpha band activity during spaceflight (Cheron et al. 2014). While it is difficult to extrapolate such functional brain findings to the present data, this EEG work provides evidence that sensorimotor neuroplastic adaptations occur during flight; thus, some of the structural brain changes we report could be linked to neuroplastic adaptations to spaceflight.

Limitations of the present work include the small sample size, which limited power for making statistical comparisons between the 12- and 6-month groups. This also precluded us from running whole-brain voxelwise comparisons between the 12- and 6-month groups. Instead, the between-group comparisons here are only for ROIs based on our previous work. We lacked structural pre- and postflight MRI data from previous missions for these astronauts, limiting the conclusions that may be drawn here, as we were not able to assess changes in brain structure from previous flights and the recovery processes in these individuals. Although the 12-month crewmembers were older than the 6-month crewmembers, the 12-month crewmembers both fell within 2 standard deviations of the mean 6-month crewmember age. The controls were also older than all of the astronauts. However, we consider this to be a strength, as the older controls would be expected to exhibit steeper declines in brain structure over time, but in most cases the astronauts showed steeper changes. Despite these limitations, this is a unique dataset which provides an important and rare preview into the effects of 1 year in space on the human brain.

In sum, 6–12 months of spaceflight resulted in an upward shift of the brain, ventricular expansion, and regional sensorimotor and cerebellar structural changes. Twelve months of spaceflight resulted in greater structural brain changes in sensorimotor, frontal, and ventricular brain regions compared with 6 months of spaceflight. The length of time between missions and prior flight experience may play a role in how spaceflight affects brain structure. All brain changes, aside from ventricular volume increases, fully recovered by 6 months after flight. The lateral ventricles showed over a 20% increase in volume for some individuals from pre- to postflight and only partial recovery by 6 months after flight. Together with our previous work (Koppelmans et al. 2016; Lee et al. 2019) and that of others (Roberts et al. 2017), these findings demonstrate spaceflight duration-dependent brain changes. That is, these structural brain changes do not plateau during flight but instead continue through 1 year in space. It is unknown whether these brain changes represent nonspecific structural atrophy, cephalad fluid shifts, and/or adaptive neuroplasticity. Nevertheless, this work provides a foundation for understanding how the brain adapts to and recovers from spaceflight, which is imperative as longer duration interplanetary missions are being planned.

## Supplementary Material

OneYear_SuppMaterials_CerebCortex_tgaa023

MovieS1_VentricularEnlargement_tgaa023

MovieS2_GMvCompression_tgaa023

## Notes

The authors wish to thank all of the astronauts who volunteered their time, without whom this project would not have been possible. The authors also wish to thank all those involved in the Open Access Series of Imaging Studies (OASIS; https://www.oasis-brains.org) and Alzheimer’s Disease Neuroimaging Initiative (ADNI; https://www.adni.loni.usc.edu) database projects.

Private sector contributions are facilitated by the Foundation for the National Institutes of Health (www.fnih.org). The grantee organization is the Northern California Institute for Research and Education, and the study is coordinated by the Alzheimer’s Therapeutic Research Institute at the University of Southern California. ADNI data are disseminated by the Laboratory for Neuro Imaging at the University of Southern California.

K.H. analyzed the GMv, CT, cerebellar, and ventricular data, created the figures and videos, and wrote the manuscript. H.M. analyzed the FW data and participated in manuscript preparation. J.L. contributed to analysis of FW data and participated in manuscript preparation. N.B. collected and analyzed data. I.S. participated in project design and software development. Y.E. collected and analyzed data. R.R. facilitated data collection. O.P. designed the FW analysis pipeline and consulted on all FW analyses. P. R.-L., S.W., J.B., A.P., and R.S. designed the project and led the interpretation and discussion of the results. All authors participated in revision of the manuscript.
