# Supplementary material for: The Impact of 6 and 12 Months in Space on Human Brain Structure and Intracranial Fluid Shifts
Source: Cereb Cortex Commun. 2020 Jun 15;1(1):tgaa023. doi: 10.1093/texcom/tgaa023 (PMC7446230; doi:10.1093/texcom/tgaa023)
Supplement: OneYear_SuppMaterials_CerebCortex_tgaa023 [file oneyear_suppmaterials_cerebcortex_tgaa023.pdf]

Supplementary Online Content:

The impact of six and twelve months in space on human brain structure and intracranial fluid shifts

K. E. Hupfeld, H. R. McGregor, J. K. Lee, N. E. Beltran, I. S. Kofman, Y. E. De Dios, P. A. Reuter-Lorenz, R. F. Riascos, O. Pasternak, S. J. Wood, J. J. Bloomberg, A. P. Mulavara, R. D. Seidler* & for the Alzheimer's Disease Neuroimaging Initiative†

*Correspondence to: rachaelseidler@ufl.edu

†Data used in preparation of this article were obtained from the Alzheimer's Disease Neuroimaging Initiative (ADNI) database (adni.loni.usc.edu). As such, the investigators within the ADNI contributed to the design and implementation of ADNI and/or provided data but did not participate in analysis or writing of this report. A complete listing of ADNI investigators can be found at: http://adni.loni.usc.edu/wp-content/uploads/how_to_apply/ADNI_Acknowledgement_List.pdf

Contents:

Figure S1. Left and Right Crus I Volume Changes with Spaceflight and Aging.

Figure S2. Correlations of Slope of Brain Changes with Inter-Mission Time.

Figure S3. Correlations of Slope of Brain Changes with Number of Previous Missions, Number of Past Flight Days, and Number of Current Flight Days.

Figure S4. Correlations Between Preflight Ventricular Volumes and Flight Experience.

Table S1. Atlas Regions Outputted by CERES Used to Create Cerebellar ROIs.

Table S2. Slopes of Brain Changes with Spaceflight and Aging.

Table S3. Assessment of Postflight Recovery Patterns.

Table S4. Correlations Between Slope of Brain Changes and Flight Experience.

Table S5. Correlations Between Preflight Ventricular Volumes and Flight Experience.

Movie S1-S2 Captions

Other Supplementary Materials for this manuscript include:

Movies S1. Ventricular Volume Increases with Flight.

Movies S2. Gray Matter Compression at the Vertex.

Figure S1. Left and Right Crus I Volume Changes with Spaceflight and Aging.

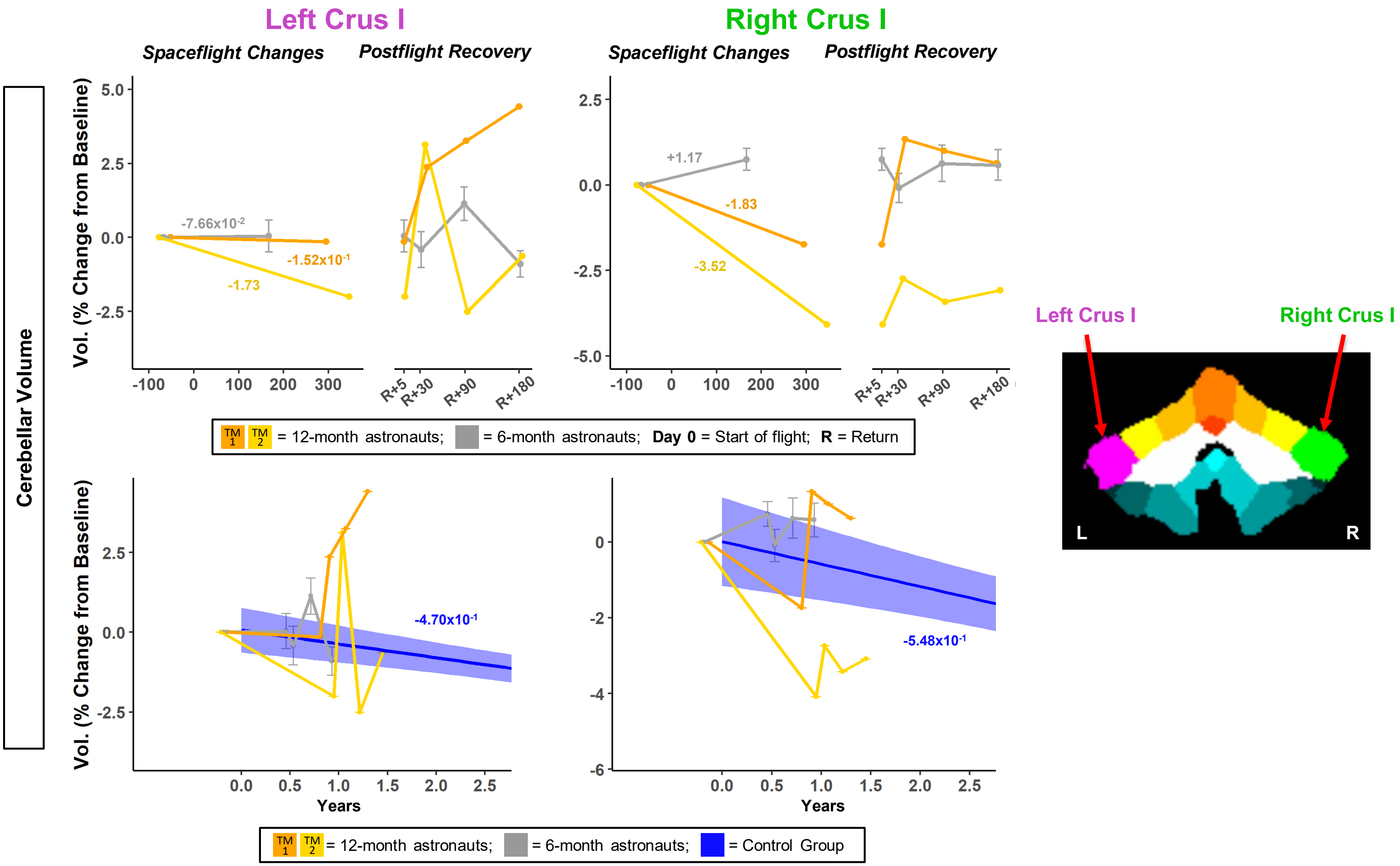

Figure S1 Legend. Cerebellar left crus I and II changes with spaceflight and recovery (*top*) and comparison to healthy aging (*bottom*). Volume changes are expressed as a percent change from baseline scan. Numeric values on the left "Spaceflight Changes" panels indicate slope of change in units of % of baseline volume per year. Error bars indicate standard error. ROIs are indicated on template in CERES space.

Figure S2. Correlations of Slope of Brain Changes with Inter-Mission Time.

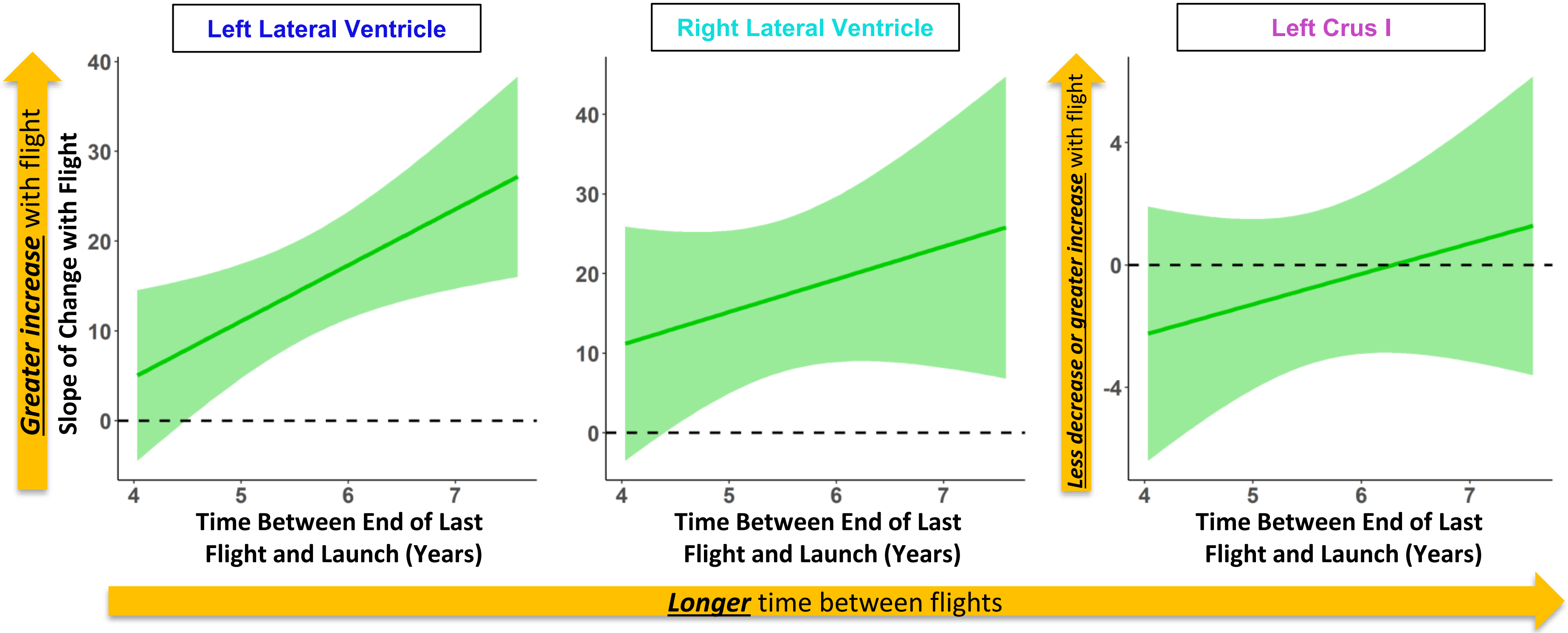

Figure S2 Legend. Graphs are shown for the three regions that showed significant correlations for this relationship. Inter-mission time is calculated as the time from the previous flight's landing day to the launch day of the present flight. Individual data points are not shown to protect astronaut privacy. For visual reference, linear model (green line) and 95% confidence interval (green shading) are shown.

Figure S3. Correlations of Slope of Brain Changes with Number of Previous Missions, Number of Past Flight Days, and Number of Current Flight Days.

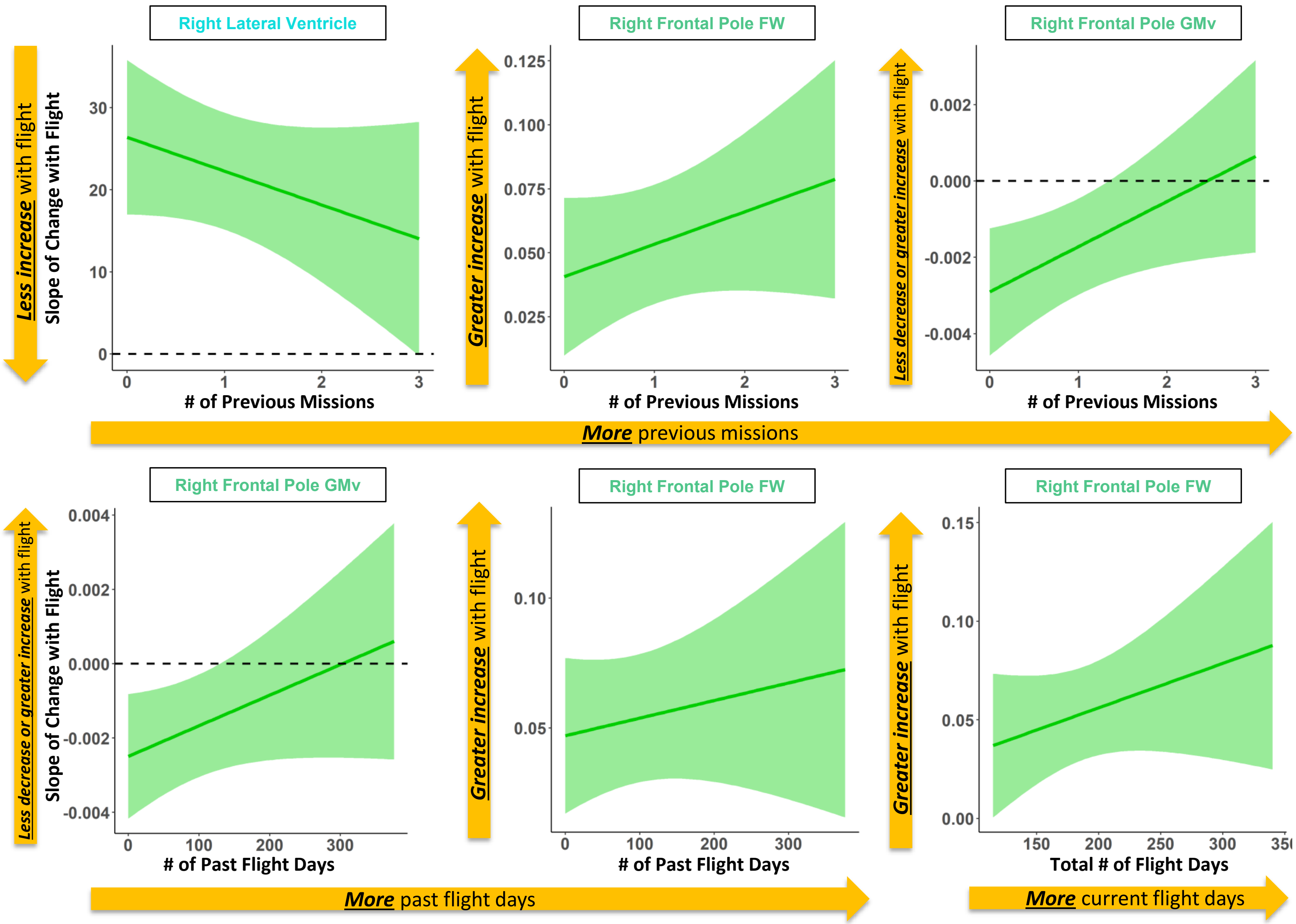

Figure S3 Legend. Graphs are shown for the three regions that showed significant correlations for these relationships. Individual data points are not shown to protect astronaut privacy. For visual reference, linear model (green line) and 95% confidence interval (green shading) are shown.

Figure S4. Correlations Between Preflight Ventricular Volumes and Flight Experience.

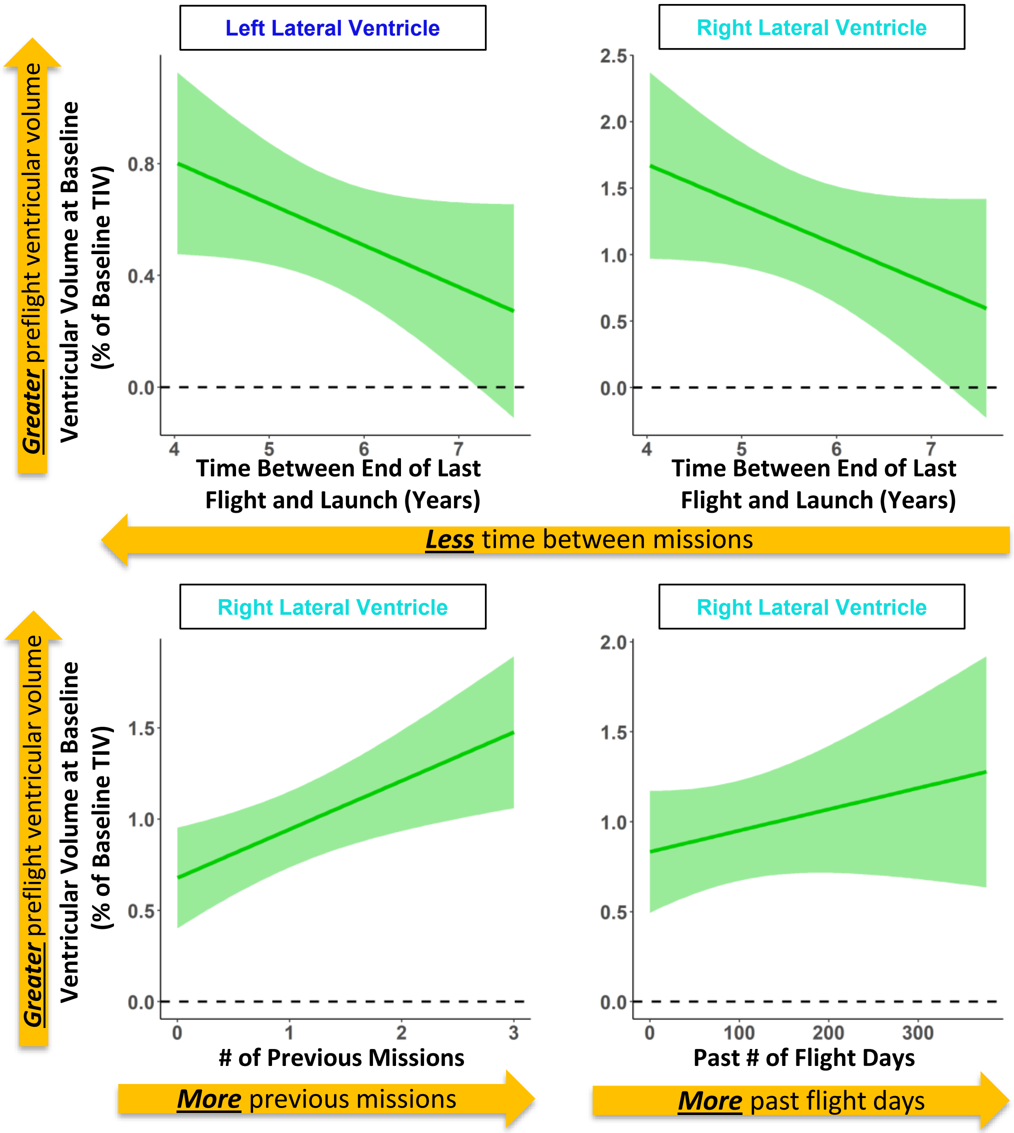

Figure S4 Legend. Graphs are shown for the four significant correlations between preflight ventricular volume and past flight experience. Individual data points are not shown to protect astronaut privacy. For visual reference, linear model (green line) and 95% confidence interval (green shading) are shown.

Table S1. Atlas Regions Outputted by CERES Used to Create Cerebellar ROIs.

Structural ROI Name	CERES Atlas Name[b]
Anterior Cerebellum	Lobule I-II
	Lobule III
	Lobule IV
	Lobule V
	Lobule VI
Posterior Cerebellum	Lobule VIIb
	Lobule VIIIa
	Lobule VIIIb
	Lobule IX
	Lobule X
	Lobule Crus II
Left Crus I[a]	Lobule Crus I, left
Right Crus I[a]	Lobule Crus I, right

[a] Total bilateral volume of each lobule (in cm^3) was used in each case, except for left and right crus I.

[b] In our past work (Bernard and Seidler 2013), we used the spatially unbiased atlas template of the cerebellum and brainstem (SUIT) toolbox (Diedrichsen and others 2009). SUIT atlas also includes the vermis, but the CERES atlas does not, so we have not included any vermal ROIs in the present work.

References

Bernard JA, Seidler RD. 2013. Relationships between regional cerebellar volume and sensorimotor and cognitive function in young and older adults. The Cerebellum 12(5):721-737.

Diedrichsen J, Balsters JH, Flavell J, Cussans E, Ramnani N. 2009. A probabilistic MR atlas of the human cerebellum. Neuroimage 46(1):39-46.

Table S2. Slopes of Brain Changes with Spaceflight and Aging.

Tests for Difference Between Slope of Brain Changes for Controls and 6-Month Astronauts[a]					12-Month Slopes of Brain Changes[b]			
	Controls (T1: *n*=20; dMRI: *n*=14)	6-Month Astronauts (*n*=10)	W	*p*	TM-1 Slope	TM-1 Slope Comparison	TM-2 Slope	TM-2 Slope Comparison
Fluid Shifts								
Left lateral ventricle								
Volume slope, median (IQR)	3.28<br>(1.07)	16.57<br>(12.28)	180	**1.62 x 10^{-4} *****	24.93	**7.6x** controls<br>**1.5x** 6-month	2.04	**0.6x** controls<br>**0.1x** 6-month
Right lateral ventricle								
Volume slope, median (IQR)	3.73<br>(2.20)	23.54<br>(6.09)	180	**1.62 x 10^{-4} *****	22.71	**6.1x** controls<br>**1.0x** 6-month	2.98	**0.8x** controls<br>**0.1x** 6-month
Right precentral gyrus								
FW slope, median (IQR)	3.00x10^{-6}<br>(2.30x10^{-5})	-2.25x10^{-2}<br>(1.01x10^{-2})	0	**1.02x 10^{-6} *****	-3.40x10^{-2}	**-11333.3x** controls<br>**1.5x** 6-month	-2.07x10^{-2}	**-6900.0x** controls<br>**0.9x** 6-month
Right postcentral gyrus								
FW slope, median (IQR)	7.00x10^{-6}<br>(3.30x10^{-5})	-2.99x10^{-2}<br>(2.33x10^{-2})	14	**5.04 x 10^{-4} *****	-5.98x10^{-2}	**-8542.9x** controls<br>**2.0x** 6-month	-3.00x10^{-2}	**-4285.7x** controls<br>**1.0x** 6-month
Right SMA								
FW slope, median (IQR)	-6.00x10^{-6}<br>(6.40x10^{-5})	-2.71x10^{-2}<br>(4.00x10^{-2})	14	**5.04 x 10^{-4} *****	-5.50x10^{-2}	**9166.7x** controls<br>**2.0x** 6-month	-2.73x10^{-2}	**4550.0x** controls<br>**1.0x** 6-month
Right frontal pole								
FW slope, median (IQR)	-2.00x10^{-6}<br>(8.50x10^{-5})	4.89x10^{-2}<br>(4.56x10^{-2})	135	**1.94 x 10^{-5} *****	6.55x10^{-2}	**-32750.0x** controls<br>**1.3x** 6-month	6.04x10^{-2}	**-30200.0x** controls<br>**1.2x** 6-month
GMv and CT Changes								
Right precentral gyrus								
GMv slope, median (IQR)	-2.27x10^{-3}<br>(2.99x10^{-3})	-1.34x10^{-2}<br>(7.66x10^{-2})	80	0.397	-8.15x10^{-2}	**35.9x** controls<br>**6.1x** 6-month	3.61x10^{-2}	**-15.9x** controls<br>**-2.7x** 6-month
CT slope, median (IQR)	3.92x10^{-3}<br>(1.56x10^{-2})	-1.24x10^{-1}<br>(1.61x10^{-1})	52	**0.035***	-2.88x10^{-1}	**-73.5x** controls<br>**2.3x** 6-month	7.13x10^{-2}	**18.2x** controls<br>**-0.6x** 6-month
Right postcentral gyrus								
GMv slope, median (IQR)	-3.30x10^{-3}<br>(1.52x10^{-3})	-2.52x10^{-2}<br>(3.87x10^{-2})	58	**0.067†**	-4.68x10^{-2}	**14.2x** controls<br>**1.9x** 6-month	-2.53x10^{-3}	**0.8x** controls<br>**0.1x** 6-month
CT slope, median (IQR)	-3.97x10^{-3}<br>(1.01x10^{-2})	-4.36x10^{-2}<br>(2.25x10^{-1})	95	0.846	-1.82x10^{-1}	**45.8x** controls<br>**4.2x** 6-month	1.77x10^{-2}	**-4.5x** controls<br>**-0.4x** 6-month
Right SMA								
GMv slope, median (IQR)	-1.94x10^{-3}<br>(1.41x10^{-3})	8.48x10^{-3}<br>(2.32x10^{-2})	147	**0.039***	1.00x10^{-2}	**-5.2x** controls<br>**1.2x** 6-month	2.19x10^{-2}	**-11.2x** controls<br>**2.6x** 6-month
Right frontal pole								
GMv slope, median (IQR)	-2.05x10^{-4}<br>(1.14x10^{-4})	-2.35x10^{-3}<br>(2.76x10^{-3})	75	0.287	-4.60x10^{-4}	**2.2x** controls<br>**0.2x** 6-month	3.58x10^{-4}	**-1.7x** controls<br>**-0.2x** 6-month
Cerebellar Volume Changes								
Anterior cerebellum								
Volume slope, median (IQR)	-4.56x10^{-1}<br>(1.01x10^{-1})	8.42x10^{-1}<br>(1.06)	180	**1.62 x 10^{-4} *****	-2.14	**4.7x** controls<br>**-2.5x** 6-month	1.86	**-4.1x** controls<br>**2.2x** 6-month
Posterior cerebellum								
Volume slope, median (IQR)	-6.00x10^{-1}<br>(2.63x10^{-1})	1.50<br>(1.90)	173	**7.54 x 10^{-4} *****	-2.06	**3.4x** controls<br>**-1.4x** 6-month	0.34	**-0.6x** controls<br>**0.2x** 6-month

Left crus I								
Volume slope, median (IQR)	-4.70x10^{-1} (2.85x10^{-1})	-7.66x10^{-2} (3.79)	131	0.183	-1.52x10^{-1}	**0.3x** controls<br>**2.0x** 6-month	-1.73	**3.7x** controls<br>**22.6x** 6-month
Right crus I								
Volume slope, median (IQR)	-5.48x10^{-1} (4.42x10^{-1})	1.17 (2.23)	170	**1.35 x 10^{-3} ****	-1.83	**3.3x** controls<br>**-1.6x** 6-month	-3.52	**6.4x** controls<br>**-3.0x** 6-month

$^{\dagger}p < 0.10$; $^{*}p < 0.05$; $^{**}p < 0.01$; $^{***}p < 0.001$. FW = free water; GMv = gray matter volume; CT = cortical thickness.

[a] The left panel presents the median slope of brain changes with normal aging, in units of change per year, for the T1 and dMRI control groups, as well as the median slope of brain changes, in units of change per year, from pre- to postflight for the 6-month astronauts. In parentheses under each median slope is the interquartile range (IQR) for that slope. W and p values are presented for the results of a nonparametric Wilcoxon signed-rank test for between-group differences in slope values.

[b] For qualitative comparison, the right panel presents the slope values for each of the 12-month astronauts and a percentage comparison to the 6-month and control groups using the formula: (12-month astronaut brain value) / (median 6-month or control group brain value).

Table S3. Assessment of Postflight Changes Over Time.

	6-Month Astronauts: Fixed Effect of Time During Postflight Period[a]				Percent Recovery by 6 Months Postflight[b]		
	**Postflight Day β**	**Postflight Day β SE**	**t**	***p***	**6-Month Astronauts, Mean (SD), %**	**TM-1, %**	**TM-2, %**
Fluid Shifts							
Left lateral ventricle							
Volume	$-1.29\text{x}10^{-2}$	$1.25\text{x}10^{-2}$	-1.04	0.308	63.84% (20.78)[c]	1.79%	37.27%
Right lateral ventricle							
Volume	$-8.75\text{x}10^{-3}$	$1.24\text{x}10^{-2}$	-0.70	0.488	54.74% (22.51)[d]	7.80%	58.76%
Right precentral gyrus							
FW	$1.14\text{x}10^{-4}$	$2.30\text{x}10^{-5}$	4.96	**$3.09 \text{ x } 10^{-5}$ *****	226.61% (169.55)[e]	100.17%	177.94%
Right postcentral gyrus							
FW	$1.57\text{x}10^{-4}$	$4.37\text{x}10^{-5}$	3.59	**$1.25 \text{ x } 10^{-3}$ *****	135.82% (64.33)[f]	79.34%	131.30%
Right SMA							
FW	$1.42\text{x}10^{-4}$	$3.24\text{x}10^{-5}$	4.39	**$1.47 \text{ x } 10^{-4}$ *****	164.46% (103.61)[g]	86.70%	146.08%
Right frontal pole							
FW	$1.63\text{x}10^{-5}$	$3.72\text{x}10^{-5}$	0.44	0.664	64.24% (44.44)[h]	73.63%	14.93%
GMv and CT Changes							
Right precentral gyrus							
GMv	$-1.79\text{x}10^{-6}$	$4.04\text{x}10^{-5}$	-0.04	0.965	56.81% (48.89)[i]	89.84%	52.81%
CT	$1.60\text{x}10^{-4}$	$1.60\text{x}10^{-4}$	1.00	0.326	33.92% (16.90)[j]	78.18%	72.76%
Right postcentral gyrus							
GMv	$-1.54\text{x}10^{-5}$	$4.41\text{x}10^{-5}$	-0.35	0.730	126.93% (165.32)[k]	60.68%	42.63%
CT	$-6.78\text{x}10^{-5}$	$1.59\text{x}10^{-4}$	-0.43	0.674	197.83% (307.11)[l]	83.08%	170.73%
Right SMA							
GMv	$-5.62\text{x}10^{-5}$	$1.68\text{x}10^{-5}$	-3.36	**$2.29 \text{ x } 10^{-3}$ *****	188.76% (182.38)[m]	*1.28%*[m]	134.43%
Right frontal pole							
GMv	$7.51\text{x}10^{-5}$	$3.59\text{x}10^{-5}$	2.09	**0.045***	180.52% (280.45)[n]	123.88%	*0.59%*[n]
Cerebellar Volume Changes							
Anterior cerebellum							
Volume	$-5.34\text{x}10^{-3}$	$1.22\text{x}10^{-3}$	-4.38	**$1.49 \text{ x } 10^{-4}$ *****	185.00% (110.84%)[o]	85.54%	60.28%
Posterior cerebellum							
Volume	$-4.56\text{x}10^{-3}$	$1.61\text{x}10^{-3}$	-2.83	**$8.43 \text{ x } 10^{-3}$ ****	139.75% (97.74)[p]	110.87%	204.30%
Left crus I							
Volume	$-4.15\text{x}10^{-3}$	$3.37\text{x}10^{-3}$	-1.23	0.227	423.01% (875.09)[q]	3176.65%	68.57%
Right crus I							
Volume	$4.76\text{x}10^{-4}$	$2.74\text{x}10^{-3}$	0.17	0.864	219.96% (260.47)[r]	136.53%	24.53%

**p* < 0.05; ***p* < 0.01; ****p* < 0.001. SE = standard error; FW = free water; GMv = gray matter volume; CT = cortical thickness.

[a] The left side of the table presents results for the linear mixed model testing for a linear recovery pattern for 6-month astronauts (i.e., including only the four postflight time points in the model and testing whether the effect of time is significantly different from 0). Degrees of freedom = 28 for each of these models.

[b] The right side of the table calculates the percent recovery at six months (i.e., at the Return+180 days postflight scan) for the 6-month astronauts and for the two 12-month astronauts. Percent recovery by six months postflight was calculated using the formula: ((Return+5 days value - Return+180 days value) / (Return+5 days value) * 100%) on the values of percentage change from baseline. 6-month astronauts for whom no recovery occurred (e.g., continued ventricular volume increase postflight) were not included in the recovery percentage calculation. Recovery values that omitted any 6-month astronauts are indicated with a superscript and described in more detail below. Italics for the 12-month recovery percentages indicate no recovery and are elaborated in the footnotes below. The 6-month astronaut who dropped out before the R+180 time point was omitted from all recovery calculations.

[c] Left lateral ventricle volume: Recovery percentage includes *n*=4 six-month astronauts who showed a pattern of recovery. *n*=5 six-month astronauts showed continued increases (+4.39 ± 3.19%) at R+180 and were not included in the recovery percentage.

[d] Right lateral ventricle volume: Recovery percentage includes *n*=5 six-month astronauts who showed a pattern of recovery. *n*=4 six-month astronauts showed continued increases (+6.87 ± 2.41%) at R+180 and were not included in the recovery percentage.

[e] Right precentral gyrus FW: Recovery percentage includes *n*=8 six-month astronauts who showed a pattern of recovery; all of these individuals and both 12-month astronauts showed recovery >100%. *n*=1 six-month astronaut showed continued decreases (-0.22%) at R+180 and was not included in the recovery percentage.

[f] Right postcentral gyrus FW: Recovery percentage includes *n*=8 six-month astronauts who showed a pattern of recovery; *n*=7 of these individuals and one of the 12-month astronauts showed recovery >100%. *n*=1 six-month astronaut showed continued increases (+4.34%) at R+180 and was not included in the recovery percentage.

[g] Right SMA FW: Recovery percentage includes *n*=7 six-month astronauts who showed a pattern of recovery; *n*=6 of these individuals and one of the 12-month astronauts showed recovery >100%. *n*=2 six-month astronauts showed continued increases (+2.94%) or decreases (-6.00%) at R+180 and were not included in the recovery percentage.

[h] Right frontal pole FW: Recovery percentage includes *n*=6 six-month astronauts who showed a pattern of recovery; *n*=1 of these individuals showed recovery >100%. *n*=3 six-month astronauts showed continued increases (+2.72%, +5.68%, +14.72%) at R+180 and were not included in the recovery percentage.

[i] Right precentral gyrus GMv: Recovery percentage includes *n*=5 six-month astronauts who showed a pattern of recovery; *n*=1 of these individuals showed recovery >100%. *n*=4 six-month astronauts showed continued increases (+0.10%, +0.59%) or continued decreases (-1.61%, -3.05%) at R+180 and were not included in the recovery percentage.

[j] Right precentral gyrus CT: Recovery percentage includes *n*=6 six-month astronauts who showed a pattern of recovery. *n*=3 six-month astronauts showed continued increases (+1.34%, +5.63%) or continued decreases (-2.39%) at R+180 and were not included in the recovery percentage.

[k] Right postcentral gyrus GMv: Recovery percentage includes *n*=7 six-month astronauts who showed a pattern of recovery; *n*=4 of these individuals showed recovery >100%. *n*=2 six-month astronauts showed continued decreases (-0.66%, -1.89%) at R+180 and were not included in the recovery percentage.

[l] Right postcentral gyrus CT: Recovery percentage includes *n*=8 six-month astronauts who showed a pattern of recovery; *n*=3 of these individuals and one of the 12-month astronauts showed recovery >100%. *n*=1 six-month astronaut showed continued decreases (-2.43%) at R+180 and was not included in the recovery percentage.

[m] Right SMA GMv: Recovery percentage includes *n*=6 six-month astronauts who showed a pattern of recovery. *n*=3 six-month astronauts showed continued increases (+0.01%) or continued decreases (-1.00%, -3.31%) at R+180 and were not included in the recovery percentage. Here, one of the 12-month astronauts showed some recovery at R+30, but then continued increases (+1.28%) from R+30 to R+180.

[n] Right frontal pole GMv: Recovery percentage includes *n*=7 six-month astronauts who showed a pattern of recovery; *n*=1 of these individuals and one of the 12-month astronauts showed recovery >100%. *n*=2 six-month astronauts showed continued increases (+3.36%) or decreases (-0.87) at R+180 and were not included in the recovery percentage. Here, one of the 12-month astronauts showed little change from pre- to postflight, but continued increase (+0.59%) from R+5 to R+180.

[o] Anterior cerebellum volume: Recovery percentage includes *n*=8 six-month astronauts who showed a pattern of recovery; *n*=6 of these individuals showed recovery >100%. *n*=1 six-month astronaut showed continued increases (+0.07%) at R+180 and was not included in the recovery percentage.

[p] Posterior cerebellum volume: Recovery percentage includes *n*=7 six-month astronauts who showed a pattern of recovery; *n*=5 of these individuals and both 12-month astronauts showed recovery >100%. *n*=2 six-month astronaut showed continued decreases (-0.65%, -0.61%) at R+180 and were not included in the recovery percentage.

[q] Left crus I volume: Recovery percentage includes *n*=8 six-month astronauts who showed a pattern of recovery; *n*=5 of these individuals and one of the 12-month astronauts showed recovery >100%. *n*=1 six-month astronaut showed continued decreases (-2.31%) at R+180 and was not included in the recovery percentage.

[r] Left crus I volume: Recovery percentage includes *n*=6 six-month astronauts who showed a pattern of recovery; *n*=3 of these individuals and one of the 12-month astronauts showed recovery >100%. *n*=3 six-month astronaut showed continued increases (+1.68%, +0.51%) or continued decreases (-1.22%) at R+180 and were not included in the recovery percentage.

Table S4. Correlations Between Slope of Brain Changes and Flight Experience.

	ρ[a]	S(DF)	*p*[b]
**Inter-mission time**[c]			
Left Lateral Ventricle, volume	0.77	8(4)	**0.103**[†]
Right Lateral Ventricle, volume	0.77	8(4)	**0.103**[†]
Left Crus I, volume	0.89	4(10)	**0.033***
**Number of previous missions**			
Right Frontal Pole, FW	0.68	92.7(10)	**0.016***
Right Frontal Pole, GMv	0.62	109.88(10)	**0.033***
Right Lateral Ventricle, volume	-0.50	427.75(10)	**0.101**[†]
**Past number of flight days**			
Right Frontal Pole, FW	0.72	81.05(10)	**0.009****
Right Frontal Pole, GMv	0.58	119.48(10)	**0.047***
**Total number of flight days (present mission)**			
Right Frontal Pole, FW	0.84	45.76(10)	**0.0006*****

[†]$p < 0.10$; *$p < 0.05$; **$p < 0.01$; ***$p < 0.001$. DF = degrees of freedom.

[a] To account for the sample size and non-normal variable distributions, nonparametric Spearman rank correlation coefficients (ρ) and test statistic values (S) are presented. Each ventricular volume, GMv, CT, and cerebellar volume analysis included all 6- and 12-month astronauts ($n = 12$), except for inter-mission time ($n = 6$), as six individuals were naïve flyers. For the FW analyses, the slope for one 6-month individual could not be calculated due to low image quality (see Methods). This person has been omitted from these analyses. Thus, $n = 11$ for all FW analyses, except for inter-mission time where $n = 5$.

[b] In this table, we present only the trending ($p \leq 0.10$) and significant ($p < 0.05$) correlations.

[c] Inter-mission time is calculated as the time from the previous flight's landing day to the launch day of the present flight.

Table S5. Correlations Between Preflight Ventricular Volumes and Flight Experience.

	ρ[a]	S(DF)	*p*[b]
**Inter-mission time[c]**			
Left Lateral Ventricle, volume	-0.94	68(4)	**0.017***
Right Lateral Ventricle, volume	-0.89	66(4)	**0.033***
**Number of previous missions**			
Right Lateral Ventricle, volume	0.74	75.5 (10)	**0.006****
**Past number of flight days**			
Right Lateral Ventricle, volume	0.63	105.6(10)	**0.028***
**Age[d]**			
Left Lateral Ventricle, volume	-0.01	288(10)	0.991
Right Lateral Ventricle, volume	0.34	190(10)	0.287

*$p < 0.05$; **$p < 0.01$.

[a] To account for the sample size and non-normal variable distributions, nonparametric Spearman rank correlation coefficients (ρ) and test statistic values (S) are presented. Each ventricular volume analysis included all 12-month and 6-month subjects ($n = 12$), except for inter-mission time ($n = 6$), as six individuals were naïve flyers.

[b] In this table, we present only the significant ($p < 0.05$) correlations.

[c] Inter-mission time is calculated as the time from the previous flight's landing day to the launch day of the present flight.

[d] We include the non-significant correlations with age here for completeness, to demonstrate that greater preflight ventricular volume did *not* correlate with older age.

Movie S1-S2 Captions

Movie S1. Ventricular Volume Increases with Flight. Clip depicts pre- versus postflight structural changes for TM-1 (i.e., the 12-month astronaut who showed substantial lateral ventricular volume increases with flight). Arrows indicate regions where ventricular volume increases are visible. Pre- and postflight structural images have been rigidly coregistered. The lack of movement of the skull bone between the two images demonstrates that this coregistration was successful and that pre- to post changes seen here are indeed brain changes and not related to poor registration or subject movement. To some observers, it might appear that these images depict a different slice for pre- and postflight scans; however, these perceived differences come from the fact that the ventricles have significantly expanded and the brain has shifted upwards within the skull with spaceflight. This has pushed on the surrounding tissue, making the pre- and postflight slices visually appear slightly different.

Movie S2. Gray Matter Compression at the Vertex. Clip depicts pre- versus postflight structural changes for TM-2 (i.e., the 12-month astronaut who showed sensorimotor gray matter volume *increases* with flight). Arrows indicate region of apparent compression of gray matter at the vertex related to postflight upward shift of the brain within the skull. Pre- and postflight structural images have been rigidly coregistered. The lack of movement of the skull bone between the two images demonstrates that this coregistration was successful and that pre- to post changes seen here are indeed brain changes and not related to poor registration or subject movement.
